# Urgency in Improving Child Health Care Workers' Awareness and Knowledge of ASD: Findings From a Cross-Sectional Study in Southwest China

**DOI:** 10.3389/fpsyt.2021.703609

**Published:** 2021-09-13

**Authors:** Yu Ma, Yan Zhou, Ye Liu, Yue Ping, Yaozhou Wang, Xiao Hu, Chenxi Zhang, Tianqi Wang, Hao Zhou

**Affiliations:** ^1^Department of Neurology, Children's Hospital of Fudan University, Shanghai, China; ^2^Guiyang Baiyun District Hospital, Guiyang, China; ^3^Department of Otolaryngology, Guizhou Provincial People's Hospital, Guiyang, China; ^4^Department of Pediatrics, Guizhou Provincial People's Hospital, Guiyang, China; ^5^Department of Neurology, Guizhou Provincial People's Hospital, Guiyang, China

**Keywords:** autism spectrum disorders, awareness, knowledge, child health care workers, China

## Abstract

**Objective:** To evaluate the levels of awareness and knowledge of ASD among child health care workers in China, we conducted a cross-sectional, questionnaire-based study to assess the participants' awareness and knowledge of ASD.

**Methods:** A total of 159 child health care workers from Southwest China participated in the survey and filled out the questionnaire.Descriptive analysis was conducted on the five parts of the questionnaire, including general knowledge, symptomology, screening and diagnosis, and intervention and treatment. Univariate analysis was used to assess impacts of the participants' basic demographic characteristics on the questionnaire scores. Multivariate analysis was used to analyze association of the participants' basic demographic characteristics and the questionnaire scores.

**Results:** Less than 15% of the participants knew that ASD is a developmental, congenital and genetic disorder. Few participants knew that the symptoms include language disorder (38.4%) and social dysfunction (29.6%). A minority of the participants knew the diagnostic criteria (22.6%) and the age for early screening (14.5%). A total of 23.9% of the participants agreed that there are no effective drugs to treat ASD, and 6.3% agreed that ASD is incurable. A number of years in practice of ≥10 (OR = 0.3249, 95% CI: 0.1080–0.9189) was the main factor related to a high questionnaire score.

**Conclusions:** Most participants had relatively low levels of awareness and knowledge of ASD, especially in terms of general knowledge as well as knowledge of intervention and treatment. Working for more than 10 years was a significant predictor of higher levels of awareness and knowledge of ASD. ASD-related training and knowledge dissemination are crucial for the early diagnosis and intervention of ASD. Child health care workers' awareness and knowledge of ASD needs to be improved to help build public awareness about ASD.

## Introduction

Autism spectrum disorder (ASD) is a developmental disorder that is mainly manifested as social and communication impairments, as well as interest restriction and repetitive behaviors (1). The prevalence of ASD is increasing year by year. In the United States, the prevalence of ASD in children aged 8 years has reached 1.85% (2). The latest estimated prevalence of ASD in 6- to 12-year-old children in China is 0.70% (3). The number of disability-adjusted life-years (DALYs) of ASD is more than 58 per 100 000 population, which causes substantial health loss across the lifespan (4). Although it is widely recognized that genetic and environmental factors are responsible for the ASD phenotype, the exact pathogenic mechanisms are still poorly understood (5). Due to phenotypic and etiological heterogeneity among individuals with ASD, the early diagnosis of ASD remains a challenge (6).

Early intervention for ASD children could enhance their communication and social skills, and improve long-term prognosis and quality of life (7). Early diagnosis is essential to promote early intervention, appropriate education planning, and the arrangement of family support services (8, 9). A meta-analysis showed that global average age at ASD diagnosis is 60.48 months (10). In China, about a third of children with ASD are diagnosed at the age of older than 3 years old (11). Late diagnosis of ASD is associated with shortage of child psychiatrists and pediatricians, inadequate basic awareness of ASD in the general public, incomprehensive early screening of ASD in community, and economic development gap between urban and rural areas (12). Early diagnosis of ASD is largely depend on high level of awareness and knowledge of ASD by clinicians and parents (13). Child health care workers' ability to recognize the signs and symptoms of ASD and respond appropriately is critical to providing the best health care for children with ASD (14, 15). However, there is a lack of research on evaluation the levels of awareness and knowledge of ASD among child health care workers in China. Thus, we aimed to conduct a cross-sectional, questionnaire-based study to assess Chinese child health care workers' awareness and knowledge of ASD in terms of their general knowledge and their knowledge of ASD symptomology, screening and diagnosis, and intervention and treatment.

## Methods

### Investigation Site and Participants

**Figure 1:**
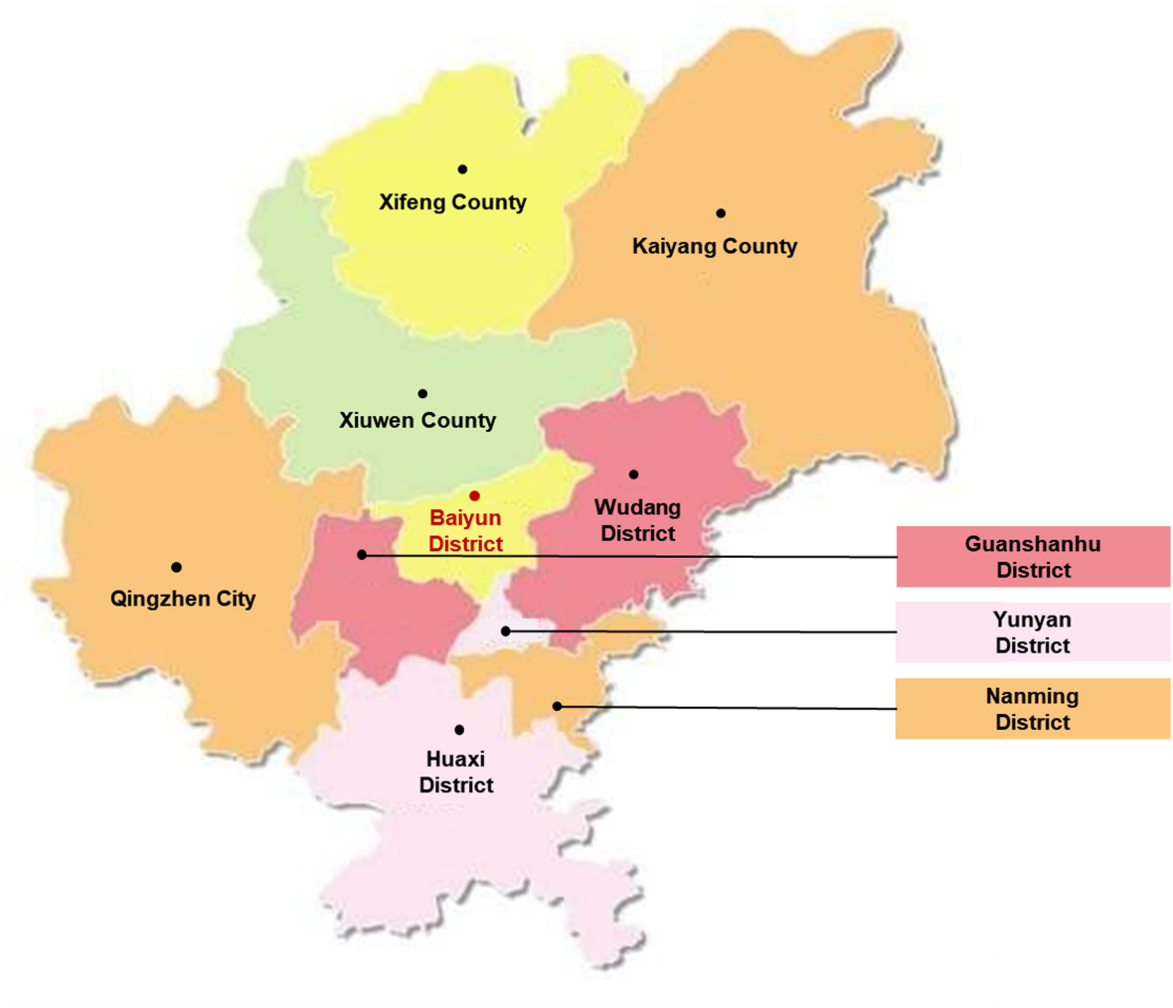
Map of Baiyun District, Guiyang City.

The study was conducted in Baiyun District, Guiyang City, Guizhou Province, China, from May 2020 to June 2020. Baiyun District (Figure 1) is the urban and rural linking area of Guiyang City, Guizhou Province, which is located in southwestern China. In 2020, the total gross domestic product (GDP) of Baiyun District was 23.678 billion yuan, which at the middle level of Guizhou province. The survey subjects were child health care workers practicing in Baiyun District.

### Questionnaire Content

The setting of questions in the questionnaire was original. The multidisciplinary team of the questionnaire development was composed of clinical experts with rich experience in children's behavioral development, psychology, epidemiology and neurological rehabilitation. The questions refer to the DSM-5 diagnostic criteria for the core symptoms of ASD, as well as the interventions recommended in the evidence-based guidelines of ASD (16, 17). The questionnaire consisted of five parts (Supplementary Table 1). The first part collected the basic demographic characteristics of the participants, including their age, gender, ethnicity, education, number of years in practice, occupation, professional title and economic income. The second part contained 6 questions that assessed the participants' general knowledge of ASD, such as whether ASD is a developmental disorder, congenital disorder or a genetic disorder. The third part contained 7 questions that evaluated their knowledge of the symptomology of ASD, including questions about special skills or interests, social withdrawal, and language barriers. 5 questions in the fourth part were about the screening and diagnosis of ASD, including diagnostic criteria and age for early screening of ASD. Knowledge of the intervention and treatment of ASD was assessed by 4 questions in the fifth part, including questions on behavioral interventions and drug therapy. All questions were multiple choice and included options for “Yes,” “No” and “I do not know.” After designing the questionnaire, we invited 20 child health care workers from the Guiyang Baiyun District Hospital to complete the questionnaire. The questions were easy understand under the health care workers' culture background, based on the feedback from these respondents.

### Survey Method

WeChat (Tencent Corp) is the most widely used social networking platform in China. The questionnaires were administered on the WeChat network using Sojump (Changsha ran Xing InfoTech Ltd). Sojump (http://www.sojump.com) is a professional online survey, evaluation and polling platform, providing personalized services including questionnaire design, data collection, customized reports and results analysis. There were totally 195 child health care workers practicing in Baiyun District, Guiyang City, Guizhou Province of China. A link to the questionnaire was sent to the WeChat group of all child health care workers. There is no time limit to fill in the questionnaire. If participants have any questions, professional staff would answer them. All questionnaires were voluntarily completed with informed consent. Questionnaires with incomplete basic information and unchecked questions were excluded.

### Data and Statistical Analysis

There were 22 questions on the questionnaire; each question was scored one point for a correct answer and no point for a not sure answer. Enumeration data are expressed as the mean ± SD, and measurement data are expressed as the constituent ratio or rate. The participants' basic demographic characteristics and their awareness of ASD in terms of general knowledge, symptoms, screening and diagnosis, and intervention treatment were analyzed by descriptive analysis.

Differences in scores by age, gender, ethnicity, education, number of years in practice, occupation, professional title and economic income were evaluated by Student's *t*-test when the distribution was normal or the Mann-Whitney U test when the distribution was skewed.

In order to study the influence factors of score, half of the total score was set as the cut-off value. Univariate analysis of the group with scores >11 and the group with scores ≤11 was conducted with the chi-square test. For sequential measures of two layers (scores >11 group and scores ≤11 group), logistic regression analysis was used to investigate the influence of age, gender, ethnicity, education, number of years in practice, occupation, professional title and economic income on the ASD awareness and knowledge scores. For the total score as a continuous variable, multiple linear regression was conducted to explore the contributions of demographic variables to total scores. Statistical analyses were performed using the SPSS statistical package program (version 20, SPSS Inc., Chicago, IL, USA), and *P* < 0.05 was considered statistically significant.

## Results

### Basic Demographic Characteristics and Professional Information of the Participants

A total of 195 child health care workers from Baiyun District, Guiyang City, Guizhou Province, China, were invited to fill in the questionnaire. Among them, 159 of participants completed the questionnaire, with the response rate of 81.5%. The basic demographic characteristics and professional information of the participants are shown in Table 1. The mean age of the participants was 32.26 ± 7.21. The male-to-female ratio was 1:8.35. The overwhelming majority of the participants were of Han ethnicity (128/159, 80.5%). Of the participants, 47.2% (75/159) had completed a college education or above. Among the participants, 27.7% were doctors, and 50.9% were nurses, with an average work experience of 8.97 ± 6.58 years.

**Table 1:** Basic demographic characteristics and professional information of the participants.

**Characteristics**	***n***	**%**
Total	159	
**Age (years)**		
<30	64	40.3
30–39	70	44.0
40–49	20	12.6
≥50	5	3.1
**Gender**		
Male	17	10.7
Female	142	89.3
**Ethnicity**		
Han	128	80.5
Minority	31	19.5
**Education**		
≤ Middle vocational	11	6.9
High vocational	73	45.9
≥College	75	47.2
**Number of years in practice**		
<10	98	61.6
10–19	47	29.6
20–29	12	7.5
≥30	2	1.3
**Occupation**		
Doctor	44	27.7
Nurse	81	50.9
Unknown	34	21.4
**Professional title**		
Primary	28	17.6
Middle	19	11.9
High	9	5.7
Unknown	103	64.8
**Economic income (RMB/month)**		
<3,000	41	25.8
3,000–4,999	52	32.7
5,000–9,999	45	28.3
≥10,000	1	0.6
Unknown	20	12.6

### Participants' Awareness and Knowledge of ASD in Children

Less than 15% of the participants knew that ASD was a developmental, congenital and genetic disorder (Table 2). Although the participants had higher levels of awareness of symptoms, a proportion of the participants lacked awareness of some of the symptoms. Only a few participants knew that the symptoms include language disorder (38.4%) and social dysfunction (29.6%) (Table 3). More than 90% of the participants were aware of the diagnosis of ASD, but a minority of the participants knew the diagnostic criteria (22.6%) and the age for early screening (14.5%) (Table 4). Regarding the intervention and treatment of ASD, 23.9% of the participants agreed that there are no effective drugs to treat ASD, and 6.3% agreed that ASD is incurable (Table 5).

**Table 2:** Participants' general knowledge of ASD in children.

**Questions on general knowledge of ASD in children**	**Correct response**	**Incorrect/ “not sure” responses**
	***n* (%)**	***n* (%)**
International Autism Day is on 2 April every year.	73 (45.9)	86 (54.1)
ASD is a common disease.	94 (59.1)	65 (40.9)
ASD is a developmental disorder.	22 (13.8)	137 (86.2)
ASD is a congenital disease.	9 (5.7)	150 (94.3)
The causes of ASD include genetic factors.	5 (3.1)	154 (96.9)
There are state subsidies for families with ASD.	41 (25.8)	118 (74.2)

**Table 3:** Participants' awareness about the symptomology of ASD in children.

**Questions on the symptomology of ASD in children**	**Correct response**	**Incorrect/“not sure” responses**
	***n* (%)**	***n* (%)**
The clinical manifestations of ASD can be mild or severe.	129 (81.1)	30 (18.9)
Children with ASD may appear to have special skills or interests in a particular aspect.	107 (67.3)	52 (32.7)
The IQ scores of children with ASD are either partially high or low or normal compared to those in the general population.	84 (52.8)	75 (47.2)
Children with ASD may be unable to speak at an age when they should be able to.	61 (38.4)	98 (61.6)
Children with ASD are indifferent to their surroundings, play alone and exhibit social withdrawal.	120 (75.5)	39 (24.5)
Children with ASD do not respond to being called names.	72 (45.3)	87 (54.7)
Children with ASD cannot use their own expressions of emotion to get your attention.	47 (29.6)	112 (70.4)

**Table 4:** Participants' awareness of screening and diagnosis of ASD in children.

**Questions on screening and diagnosis of ASD in children**	**Correct response**	**Incorrect/“not sure” responses**
	***n* (%)**	***n* (%)**
Children with ASD should go to medical institutions for consultation and treatment.	148 (93.1)	11 (6.9)
A clinic for children with ASD includes a children's neurology department, children's health department and other departments.	152 (95.6)	7 (4.4)
The DSM-5 has the latest diagnostic criteria for ASD.	36 (22.6)	123 (77.4)
Early detection and early screening are helpful to the prognosis of ASD.	146 (91.8)	13 (8.2)
The earliest age for the early screening of ASD is one year old.	23 (14.5)	136 (85.5)

**Table 5:** Participants' awareness of the intervention and treatment of ASD in children.

**Questions on the intervention and treatment of ASD in children**	**Correct response**	**Incorrect/“not sure” responses**
	***n* (%)**	***n* (%)**
There are no effective drugs to treat ASD.	38 (23.9)	121 (76.1)
Behavioral interventions for ASD include applied behavioral analysis therapy, picture vocabulary communication systems.	159 (100)	0 (0)
ASD is incurable.	10 (6.3)	149 (93.7)
Vitamin supplements during pregnancy may prevent ASD.	41 (25.8)	118 (74.2)

### Impacts of the Participants' Basic Demographic Characteristics on the Questionnaire Scores

Regarding general knowledge, the number of years in practice (*P* = 0.025) and occupation (*P* = 0.046) were the main causes of differences in scores. The number of years in practice also greatly affected the scores for intervention and treatment (*P* = 0.009). For all parts of the questionnaire, there were significant differences in the scores by the number of years in practice (*P* = 0.037) (Table 6). The average score of the whole questionnaire was 10.21 ± 3.34. A total score of ≤ 11 was considered to indicate a lack of awareness and knowledge of ASD. A total of 56 (35.2%) participants had scores > 11, and 103 (64.8%) participants had scores ≤ 11. The univariate analysis showed that occupation influenced the questionnaire scores (χ^2^ = 5.087, *P* = 0.024) (Table 7).

**Table 6:** Association of the basic demographic characteristics of the participants with their awareness and knowledge scores^a^.

**Characteristics**	***N* (%)**	**General knowledge**		**Symptomology**		**Screening/** **diagnosis**		**Intervention/** **treatment**		**All questions**	
		**Mean score ± SD**	***P* value^**b**^**	**Mean score ± SD**	***P* value^**b**^**	**Mean score ± SD**	***P* value^**b**^**	**Mean score ± SD**	***P* value^**b**^**	**Mean score ± SD**	***P* value^**b**^**
**Age (years)**											
<30	64 (40.3)	1.38 ± 1.06	0.204	3.94 ± 1.61	0.857	3.20 ± 0.89	0.704	1.53 ± 0.64	0.827	10.05 ± 3.05	0.573
≥30	95 (59.7)	1.64 ± 1.22		3.94 ± 1.82		3.16 ± 0.87		1.58 ± 0.72		10.32 ± 3.53	
**Gender**											
Male	17 (10.7)	1.65 ± 1.32	0.708	3.82 ± 1.47	0.538	3.35 ± 0.79	0.799	1.59 ± 0.80	0.993	10.41 ± 3.47	0.753
Female	142 (89.3)	1.52 ± 1.15		3.95 ± 1.77		3.15 ± 0.89		1.56 ± 0.68		10.18 ± 3.33	
**Ethnicity**											
Han	128 (80.5)	1.54 ± 1.14	0.694	3.93 ± 1.70	0.663	3.20 ± 0.86	0.530	1.59 ± 0.72	0.465	10.26 ± 3.31	0.928
Minority	31 (19.5)	1.52 ± 1.29		3.97 ± 1.89		3.06 ± 0.93		1.45 ± 0.57		10.00 ± 3.49	
**Education**											
Vocational	84 (52.8)	1.42 ± 1.12	0.231	3.80 ± 1.73	0.251	3.20 ± 0.92	0.389	1.54 ± 0.67	0.677	9.95 ± 3.35	0.445
College	75 (47.2)	1.67 ± 1.20		4.09 ± 1.73		3.15 ± 0.83		1.59 ± 0.72		10.49 ± 3.33	
**Number of years in practice**											
<10	98 (61.6)	1.35 ± 1.01	0.025^*^	3.74 ± 1.75	0.072	3.15 ± 0.99	0.749	1.44 ± 0.59	0.009^**^	9.68 ± 3.22	0.037^*^
≥10	61 (38.4)	1.84 ± 1.33		4.25 ± 1.68		3.21 ± 0.66		1.75 ± 0.79		11.05 ± 3.37	
**Occupation** ^**c**^											
Doctor	44 (35.2)	1.91 ± 1.44	0.046^*^	4.23 ± 1.79	0.082	3.07 ± 0.95	0.267	1.66 ± 0.64	0.111	10.86 ± 3.81	0.092
Nurse	81 (64.8)	1.36 ± 1.04		3.69 ± 1.82		3.19 ± 0.94		1.48 ± 0.63		9.72 ± 3.35	
**Economic income (RMB/month)** ^**c**^											
<5,000	93 (66.9)	1.48 ± 1.08	0.739	3.89 ± 1.66	0.825	3.20 ± 0.89	0.223	1.53 ± 0.64	0.280	10.11 ± 3.12	0.937
≥5,000	46 (33.1)	1.65 ± 1.40		3.93 ± 2.03		3.07 ± 0.95		1.67 ± 0.73		10.33 ± 4.07	

*a: The total score was calculated by summing the scores of each question; 1 indicates a correct response, and 0 indicates incorrect / “not sure” responses*.

*b: Bold value indicate that the difference was considered statistically significant (*

* * P < 0.05*,

** *P < 0.01)*.

*c: Unknown data were removed*.

**Table 7:** Univariate analysis of the groups with scores >11 and ≤11.

	**scores >11 (*n* = 56)**	**score ≤11 (*n* = 103)**	**χ^2^**	***P* value ^**a**^**
**Age (years)**				
<30	19	45	1.437	0.231
≥30	37	58		
**Gender**				
Male	5	12	0.282	0.596
Female	51	91		
**Ethnicity**				
Han	45	83	0.001	0.973
Minority	11	20		
**Education**				
Vocational	27	57	0.739	0.390
College	29	46		
**Number of years in practice**				
<10	29	69	3.547	0.060
≥10	27	34		
**Occupation** ^**b**^				
Doctor	22	22	5.087	0.024*
Nurse	24	57		
**Economic income (RMB/month)** ^**b**^				
<5,000	33	60	0.445	0.505
≥5,000	19	27		

a: Bold value indicate that the difference was considered statistically significant (*P < 0.05).

*b: Unknown data were removed*.

**Figure 2:**
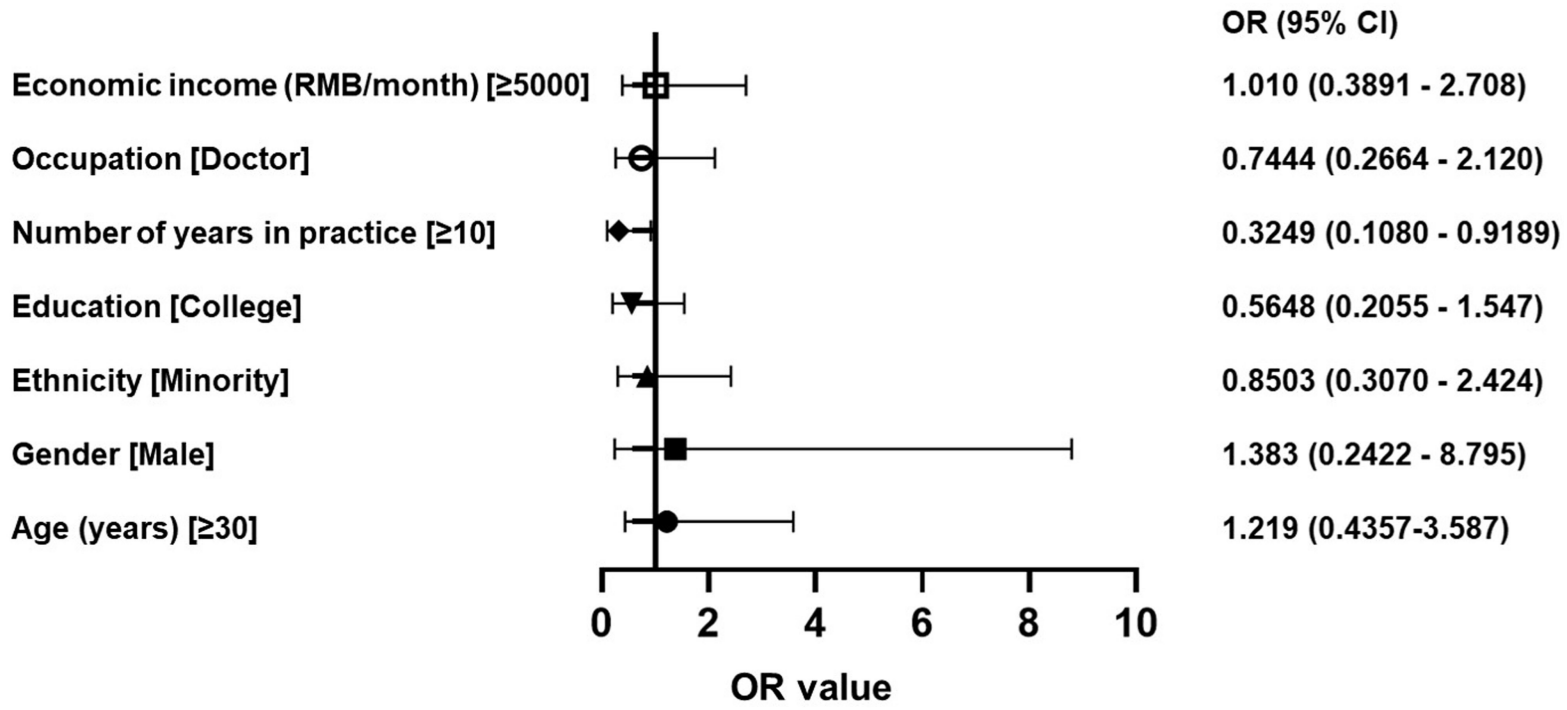
Forest plot of logistic regression.

Logistic regression was used for the multivariate regression analysis, and the results showed that a number of years in practice of ≥10 (OR = 0.3249, 95% CI: 0.1080–0.9189) was the main factor leading to high questionnaire scores (Figure 2).

Using total scores as the dependent variable and age, gender, ethnicity, education, number of years in practice, occupation and economic income as independent variables, the analysis of total scores influencing factors was performed using multiple linear regression. The results showed that number of years in practice was the factor influencing total scores (*F* = 7.284, *P* = 0.008). The results showed that number of years in practice of ≥10 contributed to the higher level of total scores (*P* = 0.008, shown in Table 8).

**Table 8:** Multiple linear regression analysis for factors influencing total scores.

	**B**	**SE (B)**	**β**	***t***	***P* value ^**a**^**	**95 % CI for B**
Age (years) (1= “ <30”, 2= “≥30”)	−1.366	0.866	−0.187	−1.577	0.118	−3.083–0.351
Gender (1= “Female”, 2= “Male”)	0.337	1.506	0.022	0.224	0.824	−2.650–3.323
Ethnicity (1= “Minority”, 2= “Han”)	0.575	0.883	0.064	0.651	0.516	−1.175–2.325
Education (1= “Vocational”, 2= “College”)	0.677	0.864	0.090	0.784	0.435	−1.035–2.390
Number of years in practice (1= “ <10”, 2= “≥10”)	2.436	0.903	0.318	2.699	0.008^*^	0.646–4.226
Occupation (1= “Nurse”, 2= “Doctor”)	0.596	0.899	0.079	0.663	0.509	−1.186–2.378
Economic income (RMB/month) (1= “ <5000”, 2= “≥5000”)	−0.103	0.825	−0.013	−0.125	0.901	−1.738–1.533

*a: Bold value indicate that the difference was considered statistically significant (*

* *P < 0.05)*.

## Discussion

The prevalence estimates of ASD in China differ significantly from those in the West, and the key factor in this difference may be the lack of awareness of ASD in China (18). Gaps in awareness and knowledge of ASD among child health care workers may lead to delays in diagnosis and intervention. Therefore, child health care workers' awareness and knowledge of ASD are crucial for the early intervention and treatment of ASD. Our study is the first to investigate awareness and knowledge of ASD among Chinese child health care workers based on an original questionnaire. Some studies used Knowledge about Childhood Autism among Health Workers (KCAHW) questionnaire, which was a useful tool for assessing health workers' baseline knowledge of ASD in children, to measure knowledge level of health workers on ASD in primary care settings (19–21). The KCAHW questionnaire is divided into four domains, including impairments in social interaction, communication, obsessive and compulsive, as well as type of disorder and possible comorbid conditions and onset (22). Compared with KCAHW questionnaire, our original questionnaire adds the questions beyond the symptomology. The assessment of our questionnaire includes general knowledge and knowledge of symptomology, screening and diagnosis, and intervention and treatment of ASD. In our study, most participants had relatively low levels of awareness and knowledge of ASD, especially in terms of general knowledge as well as knowledge of intervention and treatment.

Other reports on awareness of ASD among medical practitioners are consistent with the findings of our study. Only 44.6% of general practitioners in Pakistan had heard of ASD (23). Approximately 66.7% of family physicians had not received professional education on ASD, and 70.8% of them had not referred any child who was suspected to have ASD to a child psychiatrist in the past 6 months (24). Non-neuropsychiatric resident doctors also lacked knowledge about ASD in children (25). In addition, pharmacists were found to have gaps in their awareness and knowledge of ASD, especially in terms of etiology (26–28). These studies suggest that the lack of awareness of ASD is consistent across different countries and among different groups of health care workers.

In addition to medical practitioners, there is also a lack of awareness and knowledge of ASD in the general public. According to a report, in the Chinese population, knowledge of ASD is mainly lacking regarding the topics of core symptoms, comorbidity and prognosis (18). Parents and teachers are important members of the general public, whose cognitive level of ASD affecting the early recognition of ASD (29). In a survey of parents, 75% had heard of ASD, but the parents had poor knowledge of signs and symptoms (30). In addition, 83% of preschool teachers answered incorrectly on more than half of the questions on a questionnaire that assessed knowledge of ASD (31).

In our study, working for more than 10 years was a significant predictor of higher levels of awareness and knowledge of ASD. Similarly, Akhter et al. reported that medical workers with 11 to 15 years of practice experience had higher awareness scores, while those with 1 to 5 years had lower scores (20). As was reported, the level of awareness of ASD was also associated with a number of other factors. Compared with medical practitioners, alliance medical practitioners were found to have better knowledge of ASD (20). Pediatricians, psychiatrists and doctors working in tertiary hospitals had good knowledge of ASD, while general practitioners had poor knowledge (32).

In our study, the medical institutions in Baiyun District have a low level of medical resources and an inefficient hierarchical structure. Specialized hospitals are insufficient in number and small in scale. Therefore, the investigation of Baiyun District reflects the level of medical services in areas with poor medical care, such as Southwest China. A Chinese study showed that children with ASD who live in suburban and rural areas were diagnosed at least 6 months later than children who live in urban areas (11). Economic development gaps between urban and rural areas might contribute to differences in the knowledge of ASD. In high-income economies, increased scientific interest in the development of evidence-based interventions for ASD could help raise awareness and knowledge of ASD among the public and professionals (33).

Ninety-eight percent of parents said that the significance of an ASD diagnosis and access to support services were important, but professionals rarely provided relevant professional guidance to them (34, 35). Therefore, child health care workers' knowledge of ASD needs to be improved. Increasing the level of awareness of ASD, especially in terms of general knowledge as well as knowledge of intervention and treatment, among child health care workers is critical to early diagnosis and intervention for children with ASD.

## Limitations

The small sample size due to the restriction of the scope of the respondents may affect the generality of the study findings. The study was based on a self-completed questionnaire, which may be subject to reporting bias. We cannot exclude that only motivated medical workers were more likely to participate. Therefore, findings on awareness of ASD may be overestimated. Besides, the methods adopted for the scoring of this original questionnaire might impact the results. In our single-center study, the participants were limited to child health care workers in Baiyun District, Guiyang City, Guizhou Province, China, so the results only represent the situation in southwestern China. Further multicenter, large-scale studies are needed to better investigate the awareness and knowledge levels of ASD in China.

## Conclusions

We conducted a cross-sectional, questionnaire-based study to assess Chinese child health care workers' awareness and knowledge of ASD. We revealed that most participants had relatively low levels of awareness and knowledge of ASD, especially in terms of their general knowledge as well as their knowledge of intervention and treatment. Moreover, working for more than 10 years was a significant predictor of higher levels of awareness and knowledge of ASD. ASD-related training and knowledge dissemination are crucial for the early diagnosis and intervention of ASD. Child health care workers' awareness and knowledge of ASD needs to be improved to help build public awareness about ASD.

## Data Availability Statement

The original contributions presented in the study are included in the article/Supplementary Material, further inquiries can be directed to the corresponding author/s.

## Ethics Statement

The studies involving human participants were reviewed and approved by Institutional Ethics Committee at Guizhou Provincial People's Hospital. The participants provided their written informed consent to participate in this study.

## Author Contributions

HZ conceived of the study. YM contributed to the analysis, synthesis and interpretation of the results, and wrote the manuscript. YZ, YL, YP, YW, XH, CZ, and TW contributed to the recruitment of the participants and questionnaire collection. All authors contributed to the preparation of the manuscript.

## Funding

This project was supported by the National Natural Science Foundation of China (NSFC, 81860280).

## Conflict of Interest

The authors declare that the research was conducted in the absence of any commercial or financial relationships that could be construed as a potential conflict of interest.

## Publisher's Note

All claims expressed in this article are solely those of the authors and do not necessarily represent those of their affiliated organizations, or those of the publisher, the editors and the reviewers. Any product that may be evaluated in this article, or claim that may be made by its manufacturer, is not guaranteed or endorsed by the publisher.
