# Supplementary material for: Urgency in Improving Child Health Care Workers' Awareness and Knowledge of ASD: Findings From a Cross-Sectional Study in Southwest China
Source: Front Psychiatry. 2021 Sep 13;12:703609. doi: 10.3389/fpsyt.2021.703609 (PMC8473631; doi:10.3389/fpsyt.2021.703609)
Supplement: Supplementary file 1 [file Table_1.DOCX]

**Table S1 Autism Spectrum Disorders (ASD) Survey: Awareness and Knowledge of ASD Among** **Child Health Care Workers**

Please mark “√” in the appropriate options.

| **Basic Information** |  |  | | |  | |  | |
| --- | --- | --- | --- | --- | --- | --- | --- | --- |
| Age (years) | □<30 | □30-39 | | | □40-49 | | □≥50 | |
| Gender | □Male | □Female | | |  | |  | |
| Ethnicity | □Han | □Minority | | |  | |  | |
| Education | □≤Middle vocational | □High vocational | | | □≥College | |  | |
| Number of years in practice | □<10 | □10-19 | | | □20-29 | | □≥30 | |
| Occupation | □Doctor | □Nurse | | | □Others | |  | |
| Professional title | □Primary | □Middle | | | □High | | □Others | |
| Economic income | □<3000 | □3000-4999 | | | □5000-9999 | | □≥10000 | |
| **General Knowledge of ASD in Children** | | | Yes | No | | I do not know | |  |
| International Autism Day is on 2 April every year. | | | □ | □ | | □ | |  |
| ASD is a common disease. | | | □ | □ | | □ | |  |
| ASD is a developmental disorder. | | | □ | □ | | □ | |  |
| ASD is a congenital disease. | | | □ | □ | | □ | |  |
| The causes of ASD include genetic factors. | | | □ | □ | | □ | |  |
| There are state subsidies for families with ASD. | | | □ | □ | | □ | |  |
| **Symptomology of ASD in Children** | | | Yes | No | | I do not know | |  |
| The clinical manifestations of ASD can be mild or severe. | | | □ | □ | | □ | |  |
| Children with ASD may appear to have special skills or interests in a particular aspect. | | | □ | □ | | □ | |  |
| The IQ scores of children with ASD are either partially high or low or normal compared to those in the general population. | | | □ | □ | | □ | |  |
| Children with ASD may be unable to speak at an age when they should be able to. | | | □ | □ | | □ | |  |
| Children with ASD are indifferent to their surroundings, play alone and exhibit social withdrawal. | | | □ | □ | | □ | |  |
| Children with ASD do not respond to being called names. | | | □ | □ | | □ | |  |
| Children with ASD cannot use their own expressions of emotion to get your attention. | | | □ | □ | | □ | |  |
| **Screening and Diagnosis of ASD in Children** | | | Yes | No | | I do not know | |  |
| Children with ASD should go to medical institutions for consultation and treatment. | | | □ | □ | | □ | |  |
| A clinic for children with ASD includes a children’s neurology department, children’s health department and other departments. | | | □ | □ | | □ | |  |
| The DSM-5 has the latest diagnostic criteria for ASD. | | | □ | □ | | □ | |  |
| Early detection and early screening are helpful to the prognosis of ASD. | | | □ | □ | | □ | |  |
| The earliest age for the early screening of ASD is one year old. | | | □ | □ | | □ | |  |
| **Intervention and Treatment of ASD in Children** | | | Yes | No | | I do not know | |  |
| There are no effective drugs to treat ASD. | | | □ | □ | | □ | |  |
| Behavioral interventions for ASD include applied behavioral analysis therapy, picture vocabulary communication systems. | | | □ | □ | | □ | |  |
| ASD is incurable. | | | □ | □ | | □ | |  |
| Vitamin supplements during pregnancy may prevent ASD. | | | □ | □ | | □ | |  |
